# Conditions for maintenance of hepatocyte differentiation and function in 3D cultures

**DOI:** 10.1016/j.isci.2021.103235

**Published:** 2021-10-05

**Authors:** Niklas Handin, Evgeniya Mickols, Magnus Ölander, Jakob Rudfeldt, Kristin Blom, Frida Nyberg, Wojciech Senkowski, Jozef Urdzik, Varun Maturi, Mårten Fryknäs, Per Artursson

**Affiliations:** 1 Department of Pharmacy, Uppsala University, 75123 Uppsala, Sweden; 2 Department of Medical Sciences, Division of Cancer Pharmacology and Computational Medicine, Uppsala University, Uppsala, Sweden; 3 Biotech Research & Innovation Centre (BRIC) and Novo Nordisk Foundation Center for Stem Cell Biology (DanStem), University of Copenhagen, 2200 Copenhagen N, Denmark; 4 Department of Surgical Sciences, Uppsala University, Uppsala, Sweden

**Keywords:** Biological sciences, Proteomics, Methodology in biological sciences

## Abstract

Spheroid cultures of primary human hepatocytes (PHH) are used in studies of hepatic drug metabolism and toxicity. The cultures are maintained under different conditions, with possible confounding results. We performed an in-depth analysis of the influence of various culture conditions to find the optimal conditions for the maintenance of an *in vivo* like phenotype. The formation, protein expression, and function of PHH spheroids were followed for three weeks in a high-throughput 384-well format. Medium composition affected spheroid histology, global proteome profile, drug metabolism and drug-induced toxicity. No epithelial-mesenchymal transition was observed. Media with fasting glucose and insulin levels gave spheroids with phenotypes closest to normal PHH. The most expensive medium resulted in PHH features most divergent from that of native PHH. Our results provide a protocol for culture of healthy PHH with maintained function - a prerequisite for studies of hepatocyte homeostasis and more reproducible hepatocyte research.

## Highlights

• 3D spheroid cultures were established in 384-well format
• Eight different media variants were used to optimize the 3D cultures
• Optimized William's medium was as good as expensive commercial medium
• The 3D cultures were used to study drug metabolism and toxicity

## Graphical abstract

**Figure:**
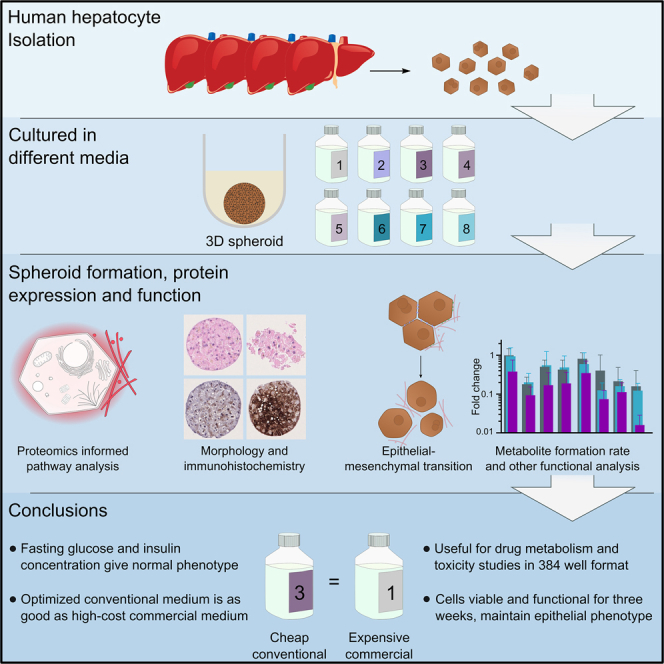

## Introduction

Hepatocytes comprise almost 80% of the liver volume, where they perform important functions including drug metabolism and xenobiotic detoxification (Kmieć, 2001; Trefts et al., 2017). Isolated primary human hepatocytes (PHH) are therefore considered the gold standard for studying hepatic metabolism and toxicity *in vitro* (Gómez-Lechón et al., 2014; Weaver et al., 2020). However, PHH are highly dependent on a finely-tuned *in vivo* microenvironment, which is difficult to reproduce *in vitro*. PHH rapidly loses their liver-specific functions in conventional 2D cultures, limiting their application to short-term studies (Bell et al., 2018).

One way to maintain long-term function of PHH is to culture grow them in 3D format (Weaver et al., 2020; Zhou et al., 2019). When 3D cultures are used, the key phenotypic traits can be maintained for a longer time time in culture. 3D cultures range from rather simple self-assemblies of PHH to more intricate co-culture systems such as scaffold-based and micro-patterned (Weaver et al., 2020; Zhou et al., 2019). Among the various 3D culture formats, PHH spheroids have been particularly popular in studies of hepatic drug metabolism and toxicity (Bell et al., 2016; Li et al., 2020; Mizoi et al., 2020). Establishment of PHH spheroid cultures is fairly straightforward. In theory, their technical reproducibility should be good given that the individual spheroids are evenly sized and formed from the same number of hepatocytes (Messner et al., 2013). However, the reproducibility of hepatocyte function varies greatly across laboratories. For instance the metabolite formation rates vary greatly (Arakawa et al., 2017; Bell et al., 2018; Foster et al., 2019). A reason could be that different culture conditions are used.

Cell culture media for PHH spheroids range from well-defined media like William's E (Bell et al., 2016; Rowe et al., 2013; Xiang et al., 2019), Dulbecco's Modified Eagle Medium/Nutrient Mixture F-12 (DMEM/F12) (Dong et al., 2016), and Advanced DMEM/F12 (Hu et al., 2018) to commercial ones such as Hepatocyte Maintenance Medium (HMM) with undisclosed contents (Buesch et al., 2018; Forsythe et al., 2018; Ölander et al., 2019). The media also vary in glucose and insulin levels, ion composition, and nutrient content. We therefore set out to investigate how such media differences influence the maintenance of PHH phenotype and liver-specific functions in 3D long-term spheroid cultures with the aim to select optimal culture conditions for drug metabolism and toxicity studies.

After establishing conditions for spheroid cultures in a 384-well format, we followed spheroid formation and phenotype using immunohistochemistry, ATP content and albumin production for three weeks. Signaling pathways and epithelial-mesenchymal transition (EMT) were investigated using quantitative global proteomics. Finally, we focused on ADME proteins relevant for drug metabolism and toxicity in functional studies. The results indicated that optimal PHH function occurs within two weeks of culture, physiological levels of glucose and insulin are preferable, and different media have a substantial impact on the culture.

## Results

### Optimizing spheroid formation in a 384-well format

**Figure 1:**
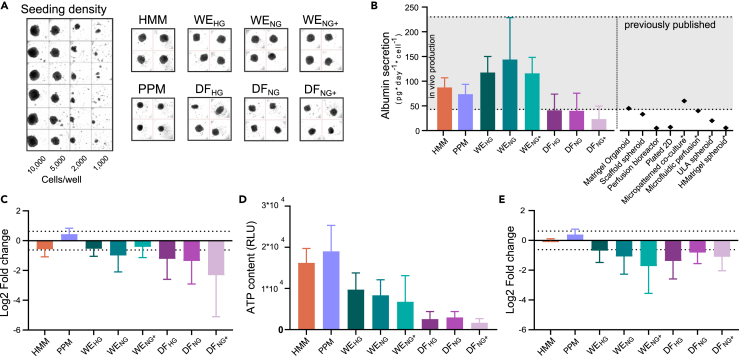
Spheroid formation and basic hepatocyte functions (A) A, left: Different seeding densities after six days in culture in HMM. A, right: Spheroid formation in the eight media (seven days in culture, seeding density of 5,000 cells/well). (B) B, left: Average albumin secretion over 3 weeks of culture. Albumin production *in vivo* ranging from dormant to maximal albumin levels (gray zone) were calculated from literature values as outlined in the methods section. B, right: albumin secretion reported from different culture models for comparison (∗marks non-human cellular origin; Table S2). (C) Average change in albumin secretion from week 1 to week 3. (D) Average ATP content over 3 weeks of culture. (E) Change in ATP content from week 1 to week 3. The span between the dotted lines in c and e indicates stable albumin and ATP levels (within 35%). b-e, Average data from four donors, error bars are showing standard deviation and media abbreviations can be found in Table 1.

To facilitate screening of drug metabolism and toxicity, the spheroid cultures were optimized for a 384-well format in commercial HMM, a medium especially developed for hepatocyte cultures. Spheroid formation was consistent using seeding densities of 5,000–10,000 cells per well, whereas spheroids with seeding densities of 2,000 and below showed a more inconsistent morphology (Figure 1A). A seeding density of 5,000 cells was chosen for all further studies to maximize oxygen and nutrient access to the interior of the spheroid.

### Culture media

Two commercial media with undisclosed content were investigated: Hepatocyte Maintenance Medium (HMM) and Cellartis Power Primary HEP Medium (PPM), as were two conventional media with well-defined contents: William's E medium (WE_HG_) and DMEM/F12 (DF_HG_; Table 1 in STAR Methods).

**Table 1:** Media supplements

	HMM	PPM	WE_HG_	WE_NG_	WE_NG+_	DF_HG_	DF_NG_	DF_NG+_
Insulin, ng/mL	10,000	≈6,000	10,000	0.58	0.58	10,000	0.58	0.58
Glucose, mg/L	≈2000^a^	≈970^a^	2000	990	990	3,151	990	990
Zinc (ZnCl_2_), μg/mL	NS	NS	NS	NS	1	NS	NS	1
Transferrin, μg/mL	5.5	NS	5.5	5.5	5.5	5.5	5.5	5.5
Selenium, ng/mL	5	NS	5	5	5	5	5	5
Dexamethasone, μM	0.1	NS	0.1	0.1	0.1	0.1	0.1	0.1
Penicillin, U/mL	100	100	100	100	100	100	100	100
Streptomycin, μg/mL	100	100	100	100	100	100	100	100
L-glutamine, mM	NS	NS	2	2	2	2	2	2

NS, Not supplemented to the medium.

a Measured values not supplied by the manufacturer.

WE_HG_ and DF_HG_ (like most conventional media) are hyperglycemic with a glucose concentration of 2,000 mg/L and an insulin concentration of 10,000 ng/mL, both far above the normal fasting state (700–1,000 mg/L for glucose and 0.19–1 ng/mL for insulin (American Diabetes Association, 2017; Williams, 2016). Therefore, we modified these media by reducing the glucose and insulin to fasting levels and named these media variants William's E normoglycemic medium (WE_NG_) and DMEM/F12 normoglycemic medium (DF_NG_), respectively (Table 1 in STAR Methods).

Because insulin is supplied in a concentrated zinc solution, the reduction of insulin solution to fasting levels also decreased the zinc concentration below physiological levels. Therefore, we modified WE_NG_ and DF_NG_ by adding back zinc ions to normal plasma levels (0.7–2.5 μg/mL) (Maxfield and Crane, 2020) and named these media variants WE_NG+_ and DF_NG+_, respectively. Thus, eight different media were investigated (Table 1 in STAR Methods).

### Albumin secretion and ATP content

The average albumin secretion was within the normal range observed *in vivo* (Figure 1B) and comparable to, or greater than, that observed in other PHH models (Davidson et al., 2016; Hu et al., 2018; Leite et al., 2016; Ong et al., 2017; Ramaiahgari et al., 2014; Toba et al., 2020; Török et al., 2011; Tostões et al., 2012). Albumin secretion was stable (defined here as within +/− 35% of first week) over time for the two commercial and the three WE-media, but lower from the spheroids cultured in the DF media (Figure 1C). No systematic differences in albumin secretion were observed between the hyper- and normo-glycemic WE and DF media.

The commercial media had spheroids with the highest ATP content, followed by the WE media. The three DF media had spheroids with the lowest ATP content of all the media (Figure 1E). For the two commercial media (HMM and PPM), the ATP content of the spheroids remained stable over the three weeks in culture (Figure 1D), whereas it was reduced two- to three-fold in the other media. As for the albumin secretion, no systematic differences could be seen between the hyper- and normoglycemic media.

### Spheroid morphology and immunohistochemistry

**Figure 2:**
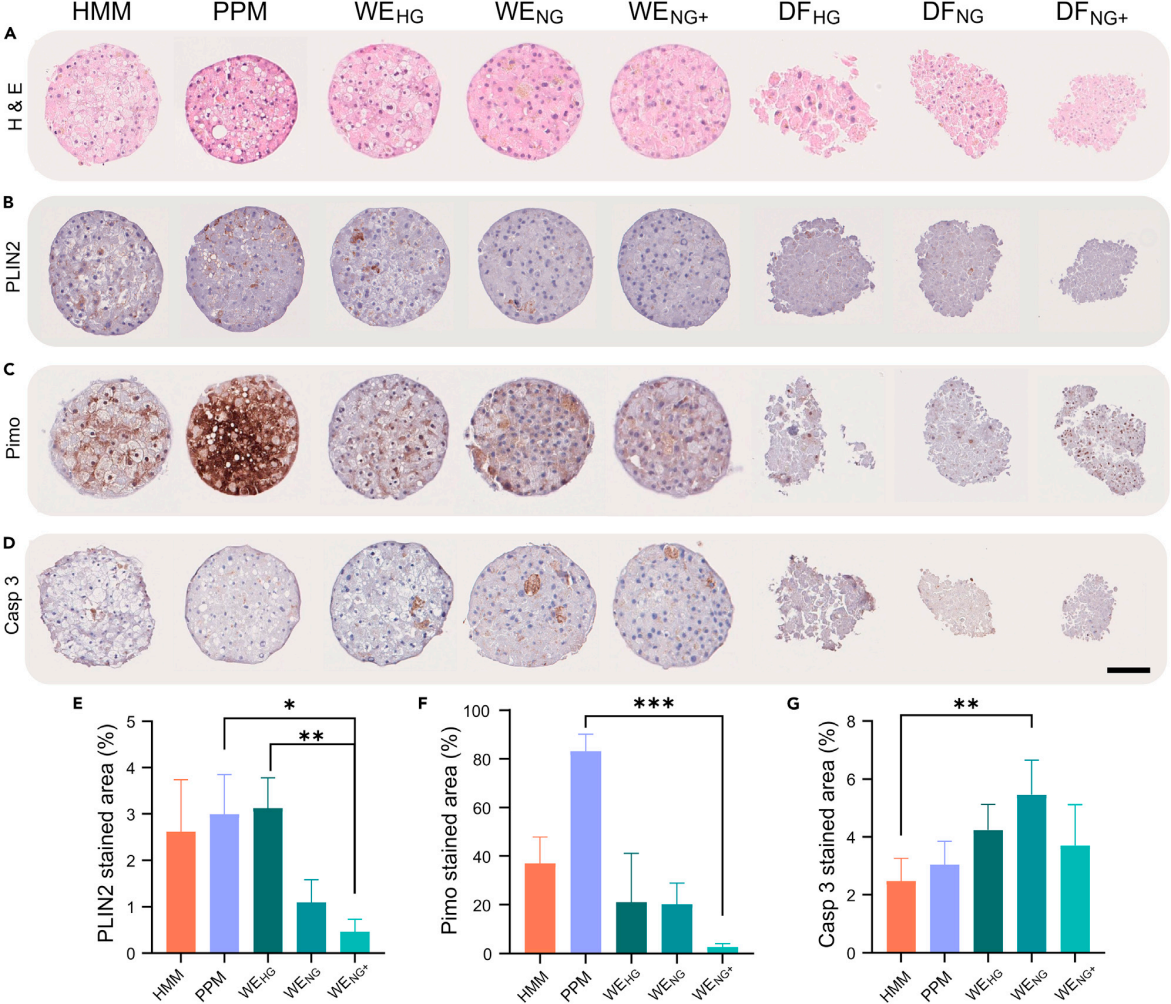
Morphology and status of PHH spheroids cultured for three weeks in different media The center of the spheroids was selected for analysis. (A) Hematoxylin and eosin staining (H & E). (B) Adipolipin (PLIN2) staining for identification of lipid deposits. (C) Pimonidazole (Pimo) staining for assessment of hypoxia. (D) Caspase 3 staining for apoptosis. (E–G) Quantification of stained areas in e, PLIN2; f, Pimo; and g, Caspase 3 (Casp 3), respectively. Five spheroids were used for quantification of average and standard deviation in each medium and images are representative for the spheroids. Scale bar = 100 μm.

We then used immunohistochemistry to allow a more detailed morphological assessment of PHH spheroids cultured in the different media. In contrast to the light microscopy (Figure 1A), the stained spheroid sections from DF media (Figure 2A) shows asymmetric and fragile 3D structures compared to those in the other media. Thus, spheroids cultivated in the two commercial and WE-media consistently formed spheroids of the expected rounded shape (Figure 2A).

In hepatocytes, hyperglycemic levels of glucose and insulin result in abnormal ectopic accumulation of lipids – a feature of steatosis (Parks, 2002). We therefore stained for adipolipin (PLIN2), a protein located around the periphery of lipid droplets, to investigate the development of a steatotic phenotype (Parry and Hodson, 2020). The PLIN2 staining confirmed the steatotic phenotype after cultivation in hyperglycemic media (Figure 2B) and its absence in normo-glycemic media (Figure 2E). Further, an increase of hollow spherical structures, typical of glycogen deposits (Aluko et al., 2020), was found in the hyperglycemic cultures (Figure 2A).

We next investigated the access to oxygen in the spheroids, because an uneven oxygen distribution could result in a hypoxic inner core, as seen in other, usually larger spheroids (Senkowski et al., 2015). For the hypoxia marker, we used the well-used pimonidazole staining (Figure 2C; (Masaki et al., 2016)). The staining was significantly higher in the PPM medium and was evenly distributed with no indication of a hypoxic core.

Because hypoxia may lead to apoptosis and/or necrosis, we also stained for the apoptosis marker caspase 3 (Figures 2D and 2G). All spheroids displayed low levels of caspase 3 with no large differences among the media (Figures 2D and 2G).

### Differences in global protein expression

**Figure 3:**
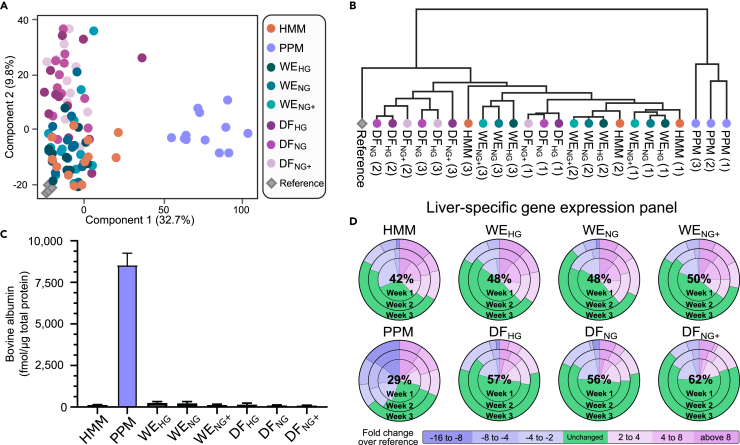
Proteome of PHH spheroids cultured in different media at one, two and three weeks of culture from four donors (A) Principal component analysis of the global proteomes for the three weeks. The number in parentheses is the percentage of variability explained by each component. (B) Hierarchical clustering of the proteomes. Each branch of the tree represents the proteome of PHH spheroids cultured in a particular medium. The culture times are given within parentheses (1, 2 or 3 weeks). Branch length is scaled to represent the Euclidean distance. (C) Abundance of bovine albumin. The protein was measured at the third week of culture using global proteomics and quantified using the total protein approach. Data are represented as average and error bars are showing standard deviation. (D) Protein expression of genes from LiGEP panel for PHH spheroids cultured in different media. Fold changes with respect to freshly thawed PHHs are color-coded as indicated in the bottom part of the figure. The average percentages of unchanged proteins (within a 2-fold change) over the three weeks are shown in the middle of each pie chart.

**Figure 4:**
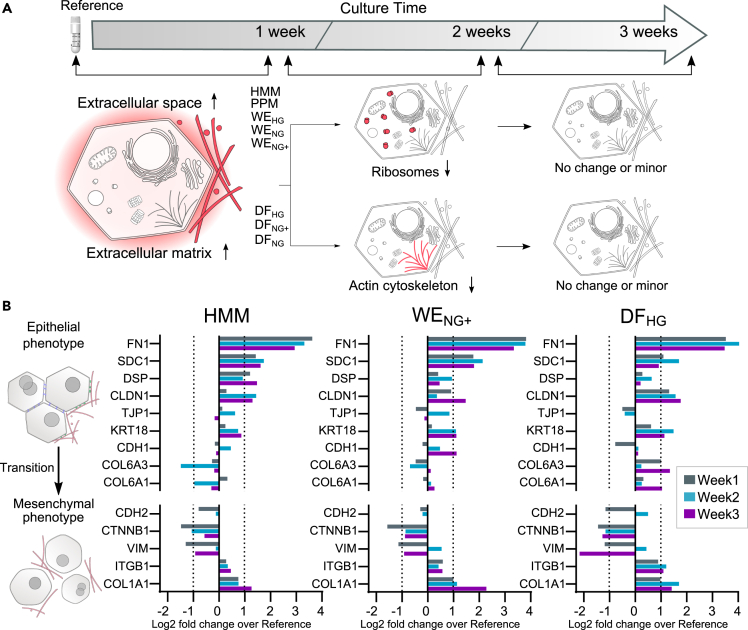
Pathway enrichment analysis and epithelial to mesenchymal transition markers assessment (A) Selection of the significantly up- and downregulated cellular components (red) from statistical enrichment analysis. Each column represents the change between two time points: cryopreserved and cultured for 1 week; cultured between 1 and 2 weeks; cultured between 2 and 3 weeks. Only cellular components with a false discovery rate below 0.05 were considered significant. See Figure S1 for all significant cellular components. (B) Expression of epithelial and mesenchymal markers (y axis) in PHH spheroids cultured in HMM, WE_NG+_ and DF_HG_ media for 3 weeks and from four donors (see Figure S4 for all media). Dotted lines indicate unchanged protein expression (±2-fold over respective reference).

**Figure 5:**
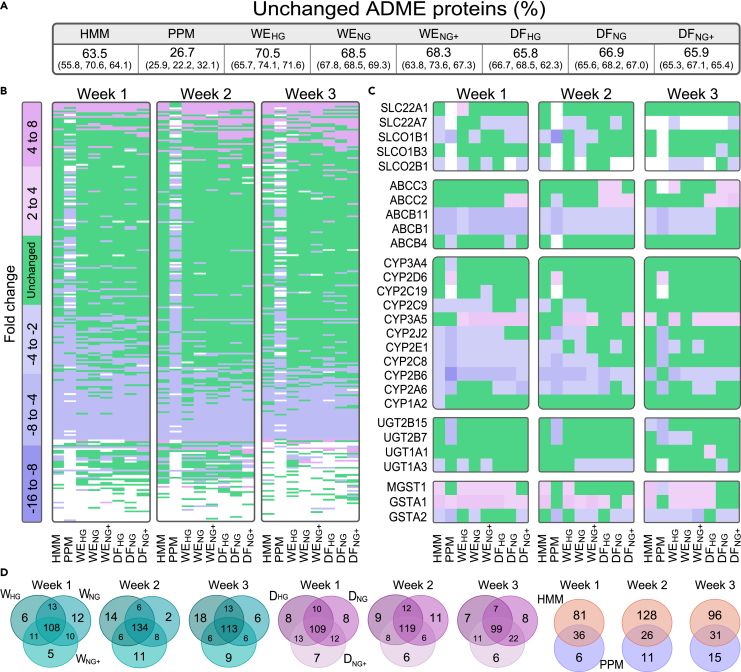
Expression of ADME-related proteins (A) Percentage of unchanged proteins for the eight different media compared to the freshly isolated PHH reference. The unchanged proteins for each week are given within parentheses (1, 2 and 3 weeks). (B) Time dependent ADME protein expression in spheroids cultured in different media. Stably expressed proteins (green) were within 2-fold of the corresponding PHH reference. (C) Time-dependent expression of clinically relevant drug transporting and drug metabolizing proteins. (D) Venn diagram of the stably expressed ADME-proteins between the hyper– and normoglycemic WE, DF, and two commercial media. Data from four different donors was used and compared to each individual reference.

**Figure 6:**
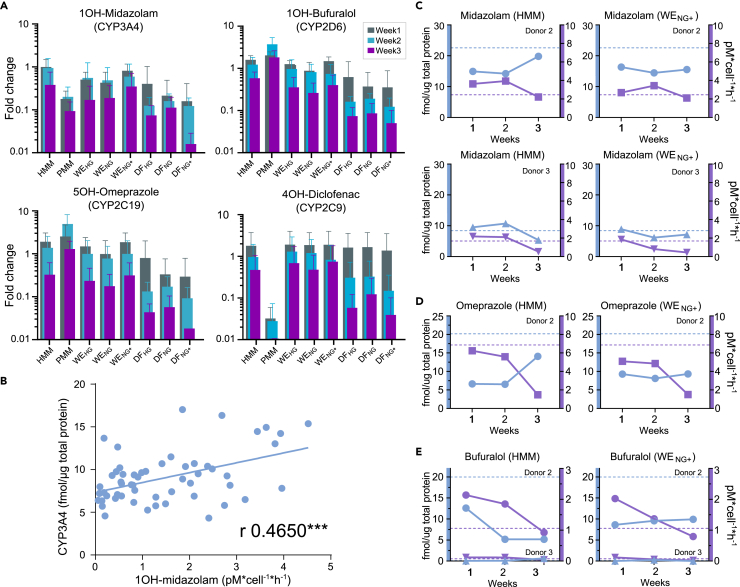
Metabolite formation and correlation with protein expression of PHH spheroids cultured in different media (A) Metabolite formation was measured using LC-MS/MS for each of the eight different media for four donors and compared to individual reference. Spheroids were cultured for one, two and three weeks. Data are represented as average and error bars are showing standard deviation. (B) Linear correlation between protein abundance and metabolite formation rate for midazolam. The PPM medium was excluded in the correlation study as it showed irregular metabolite formation rate (Figure 6A). 1OH-Midazolam showed a significant correlation with CYP3A4 abundance; Pearson's correlation coefficient is given. (C, D and E) Protein abundance (blue, CYP3A4) and metabolite formation (purple, 1OH-midazolam) for donor 2 (circles and squares) and donor 3 (triangles) from spheroids cultivated in HMM and WE_NG+_. Dashed lines indicate the value for freshly thawed PHH from the specified donor. e, shows the results from the polymorphic expression of CYP2D6 (donor 2: high expression/metabolic function, donor 3: low expression/metabolic function).

Next, we investigated the effect of the different media on the global protein expression. Freshly thawed primary human hepatocytes (PHHs) from four donors were used as references for native PHH since the proteomes of the latter are comparable to those of hepatocytes *in vivo*, e.g. (Vildhede et al., 2015). In total, 6,041 proteins were identified, of which 4,758 (79%) were quantified with razor/unique peptides of three or more. Absolute values were calculated using the total protein approach (TPA) and used for calculation of protein concentrations (Figures 3, 4, 5, 6, S1, S3–S5). The calculated TPA values can be found in Data S1, entitled “Protein concentrations.” Principal component analysis showed that the proteomes distributed into two distinct clusters along the first principal component. PHH spheroids cultured in PPM medium formed one cluster, whereas all other media formed the other cluster (Figure 3A). The proteomes were more evenly spread along the second component, making differences between media harder to distinguish.

Hierarchical clustering analysis provided additional information about the differences between the proteomes (Figure 3B). Proteomes from spheroids cultivated in WE and HMM media clustered together, indicating that their global proteomes are similar. In contrast, the proteomes from spheroids cultivated in DF media formed a separate cluster. As indicated by the principal component analysis, the spheroids cultivated in PPM had a proteome significantly different from the spheroids cultivated in the other media and freshly thawed hepatocytes. Surprisingly, we found that the PPM medium was supplemented with a large amount of bovine serum albumin (Figure 3C).

Quantification of liver specific proteins can provide additional information about the hepatocyte properties of the spheroid cultures (Ölander, et al., 2020). We therefore examined a liver-specific gene expression panel (LiGEP) of 93 genes (Kim et al., 2017) which is predominantly of hepatocyte origin (Ölander et al., 2020). Expression of the LiGEP proteins was compared in the 3D cultures and the freshly thawed hepatocytes for the four donors (Figure 3D). On average, between 42 and 62% of the LiGEP proteins had expression levels comparable to those in the freshly thawed (i.e., reference) hepatocytes. More LiGEP proteins were upregulated than in the reference. The exception was spheroids cultivated in the PPM medium, where many proteins were downregulated. The two zinc-supplemented normoglycemic media with fasting insulin levels (WE_NG+_ and DF_NG+_) had LiGEP protein expression levels more similar to the reference than did their hyperglycemic counterparts. Independently of culture conditions, LiGEP protein expression in the freshly thawed PHH differed more at one week of culture than after two and three weeks. Together, these results suggest that (except for PPM-grown cultures) once the spheroids are formed, the PHH largely regains a native expression profile.

To further investigate the time-dependent changes observed in the hierarchical clustering and in the LiGEP protein analysis, we performed a statistical enrichment analysis for cellular components. After one week in culture, proteins belonging to extracellular processes were more upregulated in the newly formed spheroids than in the freshly thawed (reference) hepatocytes, regardless of culture conditions (Figure 4A). Specifically, extracellular matrix (ECM) proteins, most likely involved in the spheroid formation, were upregulated. Examples of upregulated ECM proteins are: i) thrombospondin-1, an integrin binding protein important in tissue repair (12 fold increase; (Zhao et al., 2018); ii) beta ig-h3, a collagen and integrin binding protein involved in migration and cell adhesion (7 fold increase; (Han et al., 2016); and iii) alpha-1 and alpha 2 type I collagen (2-fold increase; (Gelse et al., 2003). In addition, extracellular space proteins were upregulated under all culture conditions. Examples of these include; i) the serum proteins plasma serine protease inhibitor, an important protein in hemostasis (6-fold increase); ii) heparin cofactor II, a coagulation factor (3-fold increase); and iii) and angiotensinogen, which regulates blood pressure, body fluid and electrolyte homeostasis (2-fold increase; (Lu et al., 2016; Nishioka et al., 1998; Rau et al., 2011; Uhlén et al., 2015).

Several other cellular components were also significantly altered during the first week of culture, probably as a result of the transition from single cell PHH suspensions to spheroids. At later time points, the significantly changed cellular components were fewer except for spheroids in DF_HG_, DF_NG_, and DF_NG+_ (Figure S1). Thus, the three DF media stood out in terms of the number of significantly changed cellular components. For instance, proteins associated with the actin cytoskeleton were downregulated in DF-cultivated spheroids (Figure 2A). For example, Arp2/3 complex, a seven-subunit protein that binds and initiates growth of new actin filaments, was downregulated 2-fold. Plastin-3 (plastins are fundamental for the formation of F-actin bundles) were also downregulated 2-fold. This downregulation of actin cytoskeleton proteins is consistent with the morphology of the PHH spheroids in the DF media (Figure 2).

During epithelial-mesenchymal transitions (EMT), cells lose their shape, structure and cell-cell adhesion and become migratory. Because the DF media caused morphological changes, we examined the expression of epithelial and mesenchymal markers (Figure 2B) (Dongre and Weinberg, 2019; Kawelke et al., 2011; Moriya et al., 2012; Regős et al., 2018; Xiang et al., 2019). In general, expression of the EMT markers was very similar across all media types. It either remained unchanged or increased over time compared to the freshly thawed (reference) PHH. The most upregulated marker was fibronectin. Plasma fibronectin is produced by hepatocytes and is the most abundant of the matrix proteins in the perisinusoidal space where it coats the hepatocytes (Wells, 2008). Although cellular fibronectin has been used as a marker for myofibroblastic differentiation for many other cell types, its absence in hepatocytes increases TGFβ signalling (Kawelke et al., 2011; Moriya et al., 2012; Wells, 2008). Other markers associated with a fibronectin-stimulated EMT (downregulation of E-cadherin, upregulation of N-cadherin and vimentin) did not change with time. Further, the mesenchymal markers were reduced or unchanged compared to the reference PHH, with the exception of alpha 1 collagen type 1, which was slightly upregulated.

### Expression of ADME-related proteins

PHH cultures are important tools in studies of drug metabolism and toxicity. Since we showed that spheroids cultivated in different media had partially different phenotypes, we performed an in-depth analysis of drug-metabolizing and other ADME-related proteins in the PHH spheroids. Of 682 ADME-related genes (Schröder et al., 2013), 315 were found in the proteomes of the various PHH spheroid cultures. This is in agreement with our previous findings (Ölander et al., 2020; Vildhede et al., 2015). The ADME-related proteins were sorted according to function using PANTHER. The two most prevalent protein classes were metabolite interconversion enzymes (46%) and transporters (16%; Figure S2). Most (63–68%) of the ADME proteins were stably expressed over time in spheroids from all media except for PPM (27%; Figure 5C). The large difference in glucose and insulin levels between hyper- and normoglycemic WE and DF media did not impact the ADME protein expression (Figure 5B). Over time, more of the ADME-related proteins were expressed at similar levels as the reference (Figure 5A). This pattern is in agreement with the time-dependent expression of the liver-specific (LiGEP) proteins in (Figure 3D).

A closer examination of transporters that influence the clinical performance of drugs (Zamek-Gliszczynski et al., 2018) showed that some expression levels were difficult to maintain over time in the 3D cultures. The clinically important protein ABCB1 (MDR1), which effluxes a broad range of drugs, had a lower expression than the reference in most media. Similarly, the bile acid transporter ABCB11 (BSEP), which is inhibited by many drugs resulting in intracellular bile acid accumulation and cholestasis, was downregulated in most media. In contrast, the ABC transporters of the MRP-family, which dispel drug and toxicant metabolites from cells, generally remained expressed at the level of the reference. A variable expression pattern was observed for the solute carrier (SLC) transporters. SLCO1B1, which transports anionic drugs such as statins into hepatocytes, was either expressed at the same level or gradually increased over time compared to reference levels. SLC22A1, which transports important cationic drugs such as metformin into the hepatocytes, was stably expressed over time in most media. However, the spheroids cultivated in PPM did not express detectable levels for most of the clinically relevant SLC transporters (Figure 5C).

The time dependent expression of the drug and chemical-metabolizing cytochrome P450 family (CYPs) also varied depending on the CYP (Figure 5C). The important CYP3A4, which metabolizes the largest fraction of drugs, was stably expressed over time at levels comparable to the reference. In contrast, the polymorphic enzyme CYP2D6 was induced in PPM media, but remained stably expressed over time in the other media. The expression of CYP2C9 and CYP2C19 increased over time to the levels of the reference. Unlike the CYPs, the UDP-glucuronosyltransferases (UGTs) were stably expressed over time at levels of the reference in most cases (Figure 5C).

The heat maps in Figures 5B and 5C summarize the tendency towards stabilization of ADME-protein expression in the different media with time. The expression patterns for clinically relevant transporters and enzymes varied from one protein to another, but after two weeks in culture, most were expressed at levels comparable to those of the reference PHHs.

### CYP metabolic function and correlation to protein expression

Because the drug metabolizing enzymes were expressed at levels similar to those in freshly thawed PHHs, we investigated their function. The four most important CYP enzymes – CYP3A4, CYP2C9, CYP2D6, and CYP2C19 (Zanger et al., 2008) – were investigated using the probe substrates midazolam (CYP3A4), bufuralol (CYP2D6), omeprazole (CYP2C19) and diclofenac (CYP2C9). The relative changes in metabolic function compared to the reference PHHs are presented in Figure 6A. In general, metabolic activity was comparable to that of the reference during the first two weeks of culture, followed by a decline during the third week. Among the WE media, the normoglycemic WE_NG+_ showed the most similarity to the reference PHHs.

At three weeks of culture, CYP protein expression stabilized (Figure 5), but the metabolic activity started to decline (Figure 6A). This reduction could not be explained by the expression levels of the CYP450 electron-donating enzymes (Figure S5). The correlation between CYP protein expression and metabolic activity was weak. A rough but significant correlation between 1OH-midazolam and CYP3A4 expression could be observed only for this metabolite/enzyme pair (Figure 6B) and none between the other metabolite/enzyme pairs (Figure S3). For the CYP3A4/1OH-midazolam pair, the donor-specific variability was greater than the difference between the media, as exemplified by HMM and WE_NG+_ (Figure 6C). For CYP2D6/1OH-bufuralol, there was a large difference in expression and function for donors 2 and 3 (Figure 6E). The low CYP2D6 expression and function in donor 3 was most likely related to the well-known genetic variability of CYP2D6 (Zhou, 2009).

### Assessment of drug-induced liver injury in different media

Recently, excellent DILI predictions were obtained for spheroids cultivated in a 96-well format with a medium similar to WE_HG_ (Vorrink et al., 2018). To compare how the best performing media influenced the DILI response, a similar approach but in the 384-well format we performed a preliminary hepatotoxicity study (Figure S6). Five prototypic compounds from the previous study were tested in HMM, WE_NG_, and WE_NG+_ media. The compounds were: one false DILI-negative compound (fluconazole), three DILI-positive compounds (diclofenac, chlorpromazine and azathioprine) and a true-negative compound (warfarin). Hepatotoxicity was evaluated after chronic exposure to each of the five drug compounds at 1×, 5×, and 20× times their clinical C_max_ concentrations for two weeks, as outlined by Vorrink et al., (2018).

The negative (warfarin) and positive (azathioprine) controls gave correct results at all concentrations in all media. One of the false negatives (fluconazole) in Vorrink et al., (2018) also gave a false negative response in WE_NG+_, while it displayed a strong DILI signal in HMM and WE_NG_. Further, diclofenac and chlorpromazine did not show a DILI signal in WE_NG+_, suggesting that the Zn-supplemented WE_NG+_ is less susceptive to the DILI compounds. These preliminary results show on clear differences between the media and motivate more comprehensive studies to identify the best performing medium for DILI predictions in the 384-well format.

## Discussion

In this paper, we investigated how commonly used culture media influence the development and function of spheroids of human hepatocytes cultivated in a 384-well screening format. To little surprise, the media with undisclosed content (HMM and PPM) were at least ten times more expensive than the disclosed media (WE and DF), with PPM being the most expensive (Table S3). The initial results showed that the four hepatocyte batches formed spheroids, and had normal albumin production and acceptable ATP levels in all eight media. However clear differences emerged when immunohistochemistry of the spheroids was analyzed.

First, the spheroids cultivated in DF varied in shape and did not hold strongly together, making them less suitable for some quantitative applications. We looked at the media compositions for possible explanations for the poorly formed spheroids and found that the DF media lack ascorbic acid. Ascorbic acid is involved in pathways important for a functioning cytoskeleton and cell junctions, e.g., collagen synthesis, actin regulation, and microtubule stabilization (Arrigoni and De Tullio, 2002; Parker et al., 2015, 2016). We believe the absence of ascorbic acid in the DF medium contributed to the poor spheroid formation. Worth mentioning is that the Advanced DMEM/F12 medium do contain ascorbic acid. However, it also includes albumin that could interfere with some applications, such as proteome analysis, metabolism and tox studies in the same ways as observed for the PPM medium.

Second, spheroids cultivated at the high glucose and insulin levels found in many standard media compositions, developed lipid droplets and increased glycogen deposits over time; these features are not present in normal healthy hepatocytes. The trend towards a steatotic phenotype was inhibited by keeping insulin and possibly glucose at physiological levels. Maintenance of a normal phenotype was further accentuated by addition of physiological zinc levels (Figure 2E). Zinc is known to affect lipid homeostasis through several pathways and to reverse alcohol induced steatosis (Kang et al., 2009).

The gradual transformation to a more steatotic phenotype is in agreement with studies that intentionally induce steatosis in spheroids (Kozyra et al., 2018). In these studies, high doses of insulin and/or free fatty acids in the culture medium upregulate genes involved in lipogenesis and downregulate gluconeogenic genes. Previous studies of PHH exposed to hyperglycemic medium and insulin in another culture format (micropatterned co-cultures) also resulted in significant hepatic lipid accumulation and decreased sensitivity to insulin-mediated inhibition of glucose output relative to a normoglycemic control (Davidson et al., 2015, 2016).

The staining of PLIN2 surrounding the lipid droplets and the formation of hollow structures typical of glycogen deposits were less apparent in our study than in previously published literature (Kozyra et al., 2018). This is probably because we did not add free fatty acids. Commonly used culture media seem to be less suitable for long-term culture of PHH spheroids because they have high glucose content and are supplemented with abnormally high levels of insulin. To maintain a normal, healthy PHH phenotype, media with fasting glucose and insulin levels should be used. Recently, glucose and insulin consumption in hepatocytes have gained interest and with a newly developed method suitable for low volume spheroid cultures (Kemas et al., 2021).

Third, when cultivated in PPM the spheroid was hypoxic. Other media also showed some hypoxic cells with the exception of the zinc-supplemented WE. Interestingly, the hypoxic cells were evenly distributed throughout the spheroids and no hypoxic core was observed as is often the case in larger spheroid cultures of e.g, cancer cells or tumours (Masaki et al., 2016; Senkowski et al., 2015). Because the components of the commercial PPM are not disclosed, it is difficult to provide an explanation for the strong Pimo staining (Figure 2). However, it is possible that commonly used medium components can cause hypoxia. As an example, the essential medium component cobalt has been implicated in hypoxia (Yuan et al., 2003). Further, this could also indicate a highly reduced environment as pimonidazole needs to be reduced for binding (Masaki et al., 2016).

The global proteome profile of the PPM spheroids deviated from the other profiles; it had the lowest expression level of liver-specific proteins. We measured high, non-physiological levels of insulin but fasting glucose levels in PPM. This medium also lacked measureable transferrin – these characteristics were not shared by the other media. Surprisingly, during the proteome analysis, we found a large non-human protein supplement corresponding to bovine albumin in the PPM medium. The undisclosed addition of such a large amount of heterologous albumin not only disturbed the proteome analysis, but may disturb metabolism and tox studies through the well-known binding properties of albumin to drugs and other chemicals (Yamasaki et al., 2013).

The global proteomes gave more detailed insights into the formation of the differentiated PHH phenotypes. During the first week in culture, the PHH forms spheroids by developing cell-cell and cell-matrix contacts. This explains the upregulation of proteins involved in these processes (Figure 4). The upregulation of serum protein production during the first week in culture could possibly be because of the absence of serum proteins in the different media (except for PPM). Upregulation of transcripts of ECM, cell-cell contact proteins and serum components such as complement and coagulation cascades are also reported elsewhere for PHH spheroid cultures (Oliva-Vilarnau et al., 2020).

Expression increased over time for proteins mediating specific hepatocyte functions although the increase was modest during the last week in culture. Interestingly, the zinc-supplemented media displayed the highest percentage of liver-specific proteins. This, in addition to significantly lower PLIN2 and Pimo staining after zinc supplementation, was unexpected. A contributing factor could be the role of zinc in insulin signaling, glucose homeostasis, and as an insulin mimetic (Myers et al., 2012; Norouzi et al., 2017). Zinc might therefore lead to better utilization of the low fasting levels of insulin by the PHH.

It has been suggested that epithelial-mesenchymal transition (EMT) inducers, like the most prominent one, TGFβ1, cause PHH dysfunctions in cell culture (Xiang et al., 2019; Zavadil and Böttinger, 2005). For instance, TGFβ signalling represses the expression of epithelial markers E-cadherin (CDH1) and the tight junction protein/Zona Occludens 1(TJP1) (Figure 4). Our EMT analysis did not show a detectable amount of the main driver TGFβ1 in the PHH spheroids. Further, CDH1 and TJP1 did not show any signs of repression in culture (Figure 4B). Absence of fibronectin increases TGFβ signaling (Kawelke et al., 2011; Moriya et al., 2012) and syndecan-1 interferes with TFGβ signalling by binding and elimination from the active site (Regős et al., 2018). The high expression of fibronectin and syndecan-1 further support the maintenance of the epithelial phenotype in the PHH spheroids (Figure 4B). In summary, the expression of epithelial markers remained essentially unchanged and mesenchymal markers did not drastically increase. Indicating that PHH spheroids remained differentiated and that no induction of EMT occurred, independent of the culture medium (Figure 4B). This is in agreement with findings indicating that EMT does not occur in 3D cultures of PHH (Wang et al., 2016).

In general, the expression of ADME proteins increased over time in parallel with the liver-specific protein panel. Clinically important xenobiotic metabolizing enzymes, such as the drug metabolizing cytochrome P450 (CYPs) and Uridine 5′-diphospho-glucuronosyltransferase (UGTs) enzymes were well expressed in most media. However, the expression varied for important drug and metabolite transporters of the SLC and ABC transporters Again, PPM cultivated PHH spheroids were outliers in that they did not express detectable amounts of most of the clinically important SLC transporters. The glucocorticoid dexamethasone included in the WE, DF and HMM media induce nuclear translocation of transcription factors that upregulate these transporters and enzymes. Although dexamethasone most likely contributed to the high expression levels of said enzymes and transporters, an in depth analysis of the complex transcriptional regulation affecting ADME protein expression was outside the scope of this article.

The expression of the metabolite efflux transporters MRP2 (ABCC2) and MRP3 (ABCC3) was comparable to that of the reference PHH. However, in most media, the low expression of other important ABC-transporters – such as the promiscuous drug efflux protein P-glycoprotein (ABCB1; MDR1) and the bile acid exporter BSEP (ABCG11) – was disappointing. Surprisingly, the two normoglycemic DF media were exceptions in that they overexpressed MRPs and expressed both P-glycoprotein and BSEP at levels comparable to the reference after three weeks in culture. Preliminary studies in our laboratory indicate that the spheroids have functional SLC and ABC transporter activity in spheroids cultivated in WE_NG+_ (data not shown). This provides the opportunity to investigate drug and chemical metabolism together with transport in the PHH spheroids.

The expression levels of the majority of the ADME proteins in the spheroids remained comparable to those of the freshly isolated reference PHH, even after two weeks of culture. However, the correlation between expression and function varies from one ADME protein to another. Differences in transcriptional regulation, access to co-factors such as NADPH, and expression and half-life of drug-metabolizing enzymes influence CYP metabolic activity, resulting in varying degrees of correlation (Neve et al., 2013; Wegler et al., 2021).

In general, the results indicated that the optimal window for using the spheroids for routine metabolism studies is during the second week in culture. However, longer incubation times may be possible if the identification of slow-forming metabolites is desirable (Bell et al., 2016). Our activity profile is comparable to, or higher than, that in a recent study on spheroids cultivated in a medium similar to WE_NG_ in a 96-well format, Figure S7 (Kanebratt et al., 2021). Interestingly, CYP3A4 enzymes are also inducible in spheroid cultures, expanding the possible applications for spheroids (Hendriks et al., 2020).

The spheroids cultured in PPM had a different metabolite formation pattern than in the other media. Two of the metabolites increased (1OH-bufuralol and 5OH-omeprazole), whereas two others (1OH-midazolam and 5OH-diclofenac) decreased compared to reference. The elevated activity of CYP2D6 and CYP2C19 in these hypoxic spheroids (Figure 6) may be related to an increased production of NAD(P)H through hypoxia-induced glycolysis. Others have also reported an increase in enzymatic activity during hypoxia (Rodríguez-Enríquez et al., 2010). The decrease in CYP3A4 and CYP2D6 metabolites in PPM can be explained by protein binding, because both midazolam and diclofenac are highly bound to albumin (Davies and Anderson, 1997; U. S. Food and Drug Administration, 2019). This confirms that media with undisclosed contents (such as the BSA in PPM) may produce confounding results.

If a drug is primarily metabolized by a single enzyme, the concentration of metabolite should ideally correlate with the expression of the enzyme. However, this is not the case (Ohtsuki et al., 2012). Metabolism patterns, also of probe molecules, are complex and often involve several parallel and/or downstream metabolic pathways. Further, probe access to the CYPs in the ER membranes can be limited by ABC-transporters. Consequently, in our study the metabolic formation rate and protein expression only correlated weakly between 1OH-midazolam and CYP3A4 expression, whereas no other correlation was significant for the other metabolite-enzyme pairs (Figure S3). To obtain better predictions of metabolite formation in spheroids, the model should take into account all factors affecting the process. A remaining complexity that will require genomic analysis is inter-individual differences caused by e.g., common genetic enzyme variants with changed function (Figure 6E).

During the course of our study, impressive results in predicting drug-induced liver injury were reported for PHH spheroids in the 96-well format (Vorrink et al., 2018). Their study detected 69% of all hepatotoxic compounds without any false positives. This exceeds both the sensitivity and specificity of all previously published *in vitro* assays. As a final functional test, we therefore performed a limited study on hepatotoxicity in our 384-format, using our three best-performing media HMM, WE_NG_, and WE_NG+_ (Figure S6). Surprisingly, spheroids cultivated in the zinc-enriched medium WE_NG+_ were unaffected by test compounds. The reason for this is unclear but potentially could be caused by zinc's antioxidant properties, metabolic homeostasis function or transcription regulatory function (Jarosz et al., 2017; Kang et al., 2009; Norouzi et al., 2017). The addition of zinc may have caused a shift in the dose-response curve, due to the beneficial effects of zinc on lipid homeostasis, hypoxia regulation, and insulin signaling. However, further testing is needed to confirm this.

In conclusion, our comprehensive comparison of commonly used cell culture media for 3D PHH spheroids revealed clear differences caused by the media. It also allowed us to identify optimal cell culture conditions for common applications such as xenobiotic metabolism and possibly also hepatotoxicity in a 384-well format. The two best-performing media (HMM and WE_NG_) gave spheroids with phenotypic features closest to those of normal PHH after two weeks in culture. WE_NG_, with fasting glucose and insulin levels, maintained a normal PHH phenotype throughout the three weeks culture period with no signs of steatotic transformation. The WE_HG_ media did not show an impaired hepatic function compared to WE_NG_, and is suitable for studying steatosis transformation and a more steatosis hepatocyte phenotype. The DF media did not form stable spheroids and also lowered CYP450 activities, while the most expensive medium, PPM, resulted in spheroid features that differ the most from the native PHH reference, resulting in unphysiological protein expression, loss of function and unnatural hypoxia staining. Even though zinc-supplemented WE_NG_ (WE_NG+_) showed fewer lipid droplets, less hypoxia staining, and slightly higher metabolite formation rate than WE_NG_, it was surprisingly resistant to prototypic hepatotoxic compounds. This finding warrants further investigation. We hope our study will contribute to a more informed and eventually more harmonized protocol for the culture of spheroids with a normal hepatocyte phenotype. A maintained healthy hepatocyte phenotype is a requirement for studies of hepatocyte homeostasis, development of disease models and reproducible studies of drug metabolism and toxicity.

### Limitations of the study

We characterize and identify differences and similarities between cell-culture media used for long-term cultivation of spheroid cultures of primary human hepatocytes. For this purpose, we use a large number of techniques for time dependent characterization of spheroid development, ranging from global proteomic to functional tests. Our key finding is that some standard media perform as well as or better than expensive commercial media. Although comprehensive, our study does not cover all possible media additives. A limitation of our study is therefore that additional media variants with possible advantageous additives exist that are not covered in our manuscript. Another limitation is that we do not follow up all of the interesting findings in this paper. These include the possible effect of ascorbic acid on the cytoskeleton and spheroid stability and the seemingly protective effect of zinc addition for some DILI compounds. Although we intend to follow up some of these observations separately ourselves, our principle is to share important preliminary observations with our research community to open up for further analysis by others than ourselves.

## STAR★Methods

### Key resources table

**Table:** 

REAGENT or RESOURCE	SOURCE	IDENTIFIER
**Antibodies**		

Rabbit caspase-3	Cell Signaling Technology	#9664S; RRID:AB_2070042
Mouse Ki-67 Antigen	Agilent	#IR626; RRID:AB_2890068
Rabbit	Cell Marque	#393A;

**Biological samples**		

Histologically normal liver tissues	Department of Surgery at Uppsala University Hospital	

**Chemicals, peptides, and recombinant proteins**		

Custom-made William’s E and DMEM/F12 without glucose, L-glutamine and phenol red	PAN-Biotech GmbH	
Hepatocyte-maintenance medium	Lonza	#CC-3197
Cellartis Power Primary HEP Medium	TaKaRa Bio	Y20020

**Critical commercial assays**		

Pimonidazole staining kit	Hypoxyprobe	Hypoxyprobe Kit
CellTiter-Glo3D assay	Promega	#G9682
human albumin ELISA kit	Thermo Fisher Scientific	#EHALB

**Deposited data**		

Global proteomics data	ProteomeXchange Consortium	ProteomeXchange: PXD024632

**Software and algorithms**		

MaxQuant version 1.6.10.43	(Cox and Mann, 2008)	https://www.maxquant.org/
UniProtKB	(The UniProt Consortium, 2021)	https://www.uniprot.org/

### Resource availability

#### Lead contact

Further information and requests for resources and reagents should be directed to and will be fulfilled by the lead contact, Per Artursson (Per.Artursson@farmaci.uu.se).

#### Materials availability

This study did not generate new unique reagents.

### Experimental model and subject details

Liver tissues were obtained from cancer patients (exclusion criterion primary liver cancer) undergoing liver resections at the Department of Surgery at Uppsala University Hospital, Sweden. The collected tissues were surplus liver tissues, surgically removed and histologically normal. Tissue donors signed an informed consent, under the ethical approval from Uppsala Regional Ethical Review Board (Ethical Approval no. 2009/028, amended 2018/1108). Patient Data for the individual donors can be found in Table S4.

### Method details

#### Primary human hepatocytes isolation

The two-step collagenase-perfusion procedure for the hepatocyte isolation was used with small modifications (LeCluyse and Alexandre, 2010). In brief, the liver tissue was rinsed from excessive blood using Hypothermosol FRS (Biolife Solutions), cannulated, and perfused with collagenase and protease buffers to release cells from the tissue matrix. For cryopreservation, the hepatocytes were resuspended in CryoStor CS10 solution (BioLife Solutions) supplemented with 10% fetal bovine serum and deep-frozen in cryovials using isopropanol-assisted controlled freezing at -80°C for 3 h. The cells were then stored at -150 °C until use.

#### Hepatocyte culture

Primary human hepatocytes from four randomly selected donors were used throughout the study, unless otherwise stated. Hepatocyte culture was performed essentially as described previously (Ölander et al., 2019). In brief, the cryopreserved hepatocytes were gently thawed and the cell suspension transferred to a thawing medium consisting of 27% isotonic Percoll (GE Healthcare) in DMEM. The cell suspension was centrifuged at 100 x g for 10 min, the supernatant aspirated, and the hepatocytes resuspended in warm suspension and attachment medium (SAM) (LeCluyse and Alexandre, 2010). The cells were seeded in ultra-low attachment 384-well plates (Corning) which were then centrifuged at 100 x g for 5 min to sediment the cells. Plates were thereafter incubated at 37°C in 5% CO2.

Two days after seeding, 80% of the medium was exchanged for one of the eight test media, see Table 1. The media were changed every 48-72 hours throughout the three weeks of culture. Samples from the cultures were collected on days 7, 14 and 21, unless otherwise stated. Shorter time points were not used because the spheroids need 3-6 days for complete formation.

#### Glucose measurement

The undisclosed glucose content of the commercial media was measured with the Precision XceedPro (Abbott, Chicago, Illinois) and the Precision PXP glucose sticks (Abbott, Chicago, Illinois), according to the manufacturer’s instructions. The measurements were performed in triplicate and the average of the values used.

#### Proteomic analysis

Proteomics analysis was performed to assess the influence of the media on global, liver-specific, and ADME protein expression over time. Once a week for three weeks, eight spheroids from each of the four donors, were pooled together, resulting in samples of 40,000 cells. Freshly thawed PHH from the corresponding batches were used as references. The sample preparation was performed as described previously (Ölander et al., 2019). Briefly, the samples were lysed and the final concentration of the lysates contained 50mM DTT and 2% SDS in 100mM Tris/HCL at pH 7.8. Protein concentrations were quantified using the tryptophan fluorescence method (Wiśniewski and Gaugaz, 2015), and sequential proteolytic digestion with trypsin and LysC was performed using the MED-FASP protocol (Wiśniewski and Mann, 2012). The peptides were separated on an easy-spray C18 reverse phase column (50 cm long, 75 μm inner diameter) on a 145-min water/acetonitrile gradient. Eluted peptides where analysed using the TopN method (full MS followed by ddMS2 scans) on an Orbitrap Q Exactive HF mass spectrometer (Thermo Fisher Scientific). The mass spec data were analysed (MaxQuant version 1.6.10.43) with the complete human proteome extracted from UniProtKB (October 2019). A detailed description of the parameters used for peptide identification and integration by MaxQuant can be found in in the deposited data. For the determination of protein abundance, the total protein approach (TPA) was used (Wiśniewski and Rakus, 2014). The mass spectrometry proteomics data have been deposited to the ProteomeXchange Consortium via the PRIDE (Vizcaíno et al., 2016) partner repository with the dataset identifier PXD024632 and quantified TPA data is also found in Data S1.

#### Global proteomics data analysis

Principal component analysis and general data handling of the proteomics data was done in Perseus version 1.6.6.0 (Tyanova et al., 2016). Figures were made in GraphPad Prism version 8.1.0. Hierarchical clustering was made in Perseus using Euclidean distances of averaged values. Statistical enrichment analysis was performed using the PANTHER classification tool (Mi et al., 2019). For each medium, the UniProt IDs and the log2 fold-change in protein concentrations compared to freshly thawed hepatocytes was uploaded to PANTHER. The PANTHER GO-Slim Cellular Component database was used to annotate enriched cellular component clusters. Clusters with false discovery rate lower than 0.05 were deemed significantly up- or downregulated.

#### Assessment of the ATP content

ATP conent (expressed as relative light units; RLU) was used to measure the energy status of the PHH spheroids. The spheroids were incubated in AccuMax cell dissociation solution (Thermo Fisher Scientific) for 30 min at 37°C followed by centrifugation at 100 x g for 5 min. The CellTiter-Glo3D assay (Promega, Madison, Wisconsin) was used, with slight modifications. Briefly, the plate was first left for 30 min at room temperature for temperature equilibration. Thereafter CellTiter-Glo® 3D was added, and the plate left in room temperature for another 20 min on a horizontal plate shaker. The luminescence was measured on a Spark plate reader (TECAN). The results are presented as mean values +/- SD of eight technical replicates performed on three independent occasions.

#### Albumin secretion

Albumin secretion (expressed as pg/cell/day) was used to measure liver function of the PHH spheroids. Medium from four wells was collected and stored at −80°C until analysis. Human albumin concentration was quantified using a human albumin ELISA kit (Thermo Fisher Scientific) according to the manufacturer's instructions. The *in vivo* production span of human albumin was calculated to be 43 pg/day/cell/(dormancy production) and 230 pg/day/cell during maximal production, using 9 g/day as dormancy level; 48 g/day as maximum level (Bernardi et al., 2012); and 139 million cells/g liver (Sohlenius-Sternbeck, 2006); and a liver weight of 1.5 kg (Molina and DiMaio, 2012). The results are presented as mean values +/− SD, unless otherwise stated.

#### Immunohistochemistry

Immunohistochemistry was performed to assess the morphology of spheroids and differentially localized processes (such as hypoxia, apoptosis and lipid markers). PHH spheroids were prepared using a standard protocol. Briefly, for hypoxia staining, spheroids were first incubated with 200 μM pimonidazole for 1h before fixation. Otherwise, three-week-old PHH spheroids were washed three times in PBS and fixed in 4% formaldehyde in PBS overnight. After fixation, the spheroids were embedded in paraffin and sectioned at a thickness of three μm. DAKO's PT Linker was used for antigen retrieval. Staining was performed using DAKO's Autostainer Link 48 and DAKO’s EnVision FLEX kit. Slides were incubated with Flex Peroxidase Block for 5 min followed by incubation with primary antibodies for 30 min. The following primary antibody dilutions were used: caspase-3 (1:100), Ki-67 (pre-diluted), pimonidazole (1:50), and Plin2 (1:200). The slides were then incubated with FLEX/HRP for 20 min, followed by incubation in FLEX DAB+ Sub-Chromo solution for 10 min and counterstained with Mayer's hematoxylin from Histolab (Spånga, Sweden) for 5 min. Then slides were rinsed with 1:5 diluted (from saturated solution) lithium carbonate water for 1 min, dehydrated using ethanol/xylene solution, and covered with Pertex from Histolab (Spånga, Sweden) using an automated glass coverslipper. The slides were then scanned using the automated scanning system Nanozoomer S60 (Hamamatsu; Sunayama-cho, Japan) and high-resolution NDPI images taken.

#### Immunohistochemistry quantification

The images of the spheroids were converted to tiff files using open source cross-platform software NDPITools (Deroulers et al., 2013) and subsequently analysed using ImageJ imaging analysis program (Rueden et al., 2017). Quotients of positively stained areas (coloured in brown) were calculated in pixels using a colour threshold adjustment method and normalized with spheroid areas also measured in pixels. Colour threshold was kept consistent between all samples. The results are expressed as a mean value of quotients of positively stained areas measured on five independent spheroids cultured in the same media, unless otherwise stated, ± S.D.

#### CYP metabolite formation

Metabolite formation rate was measured to assess the metabolic capacity of the PHH spheroids cultured in the different media. Freshly thawed PHHs in suspension were used as reference. The spheroids were incubated for 5 h at 37°C with a cocktail of prototypical cytochrome P450 substrates: 5 μM bufuralol (CYP2D6), 5 μM diclofenac (CYP2C9), 5 μM midazolam (CYP3A4) and 10 μM omeprazole (CYP2C19) in HBSS. Medium from 8 spheroids per donor was collected, pooled, and stored at −80°C until analysis.

#### Metabolite quantification

The samples were diluted with ice-cold acetonitrile/water (60:40) containing 50 nM warfarin as internal standard. The primary metabolites (1-hydroxybufuralol, 4-hydroxydiclofenac, 1-hydroxymidazolam and 5-hydroxyomeprazole) were measured using an Acquity UPLC (Waters Corp, Milford, Massachusetts) with a C18 column connected to a SCIEX Q-TRAP 6500 (Framingham, Massachusetts). The transitions are found in Table S1. Peaks were integrated using MultiQuant software version 3.0.5373.0 with a Gaussian smooth width of 1 and linear regression with weighting of concentration.- The results are presented as mean values +/− SD from the individual donors as well as compared to the reference.

#### Hepatotoxicity

Preliminary studies of hepatotoxicity were conducted as previously described for the 96-well format (Vorrink et al., 2018). Briefly, PHHs were cultured in 384-well plates in HMM, WENG or WENG+ media for 1 week to form spheroids. The compounds were dissolved in dimethyl sulfoxide (DMSO), and added to the wells at a final DMSO concentration of 0.5%. As in the Vorrink et al. study, final concentrations of 1×, 5×, and 20× times the clinical Cmax of the compounds were used The exposure medium was changed every 48–72h for the 14 days of treatment. On the last day, PHH viability was assessed by measurement of ATP content (see above) and a viability below 80% of control levels was considered hepatotoxic (Vorrink et al., 2018).

### Quantification and statistical analysis

All analyses and cteated plots were carried out using GraphPad Prism version 8.1.0, unless otherwise stated. All results are presented as mean values +/− SD, unless otherwise stated. Number of replicates can be found in each figure legend.

## Data Availability

• The mass spectrometry proteomics data have been deposited at ProteomeXchange Consortium via the PRIDE partner repository and are publicly available as of the date of publication. Accession numbers are listed in the key resources table. • This paper does not report original code. • Any additional information required to reanalyse the data reported in this paper is available from the lead contact upon request.

## Supplemental information

Document S1. Figures S1–S7 and Tables S1–S4

Data S1. Protein concentrations
