# Supplementary material for: Conditions for maintenance of hepatocyte differentiation and function in 3D cultures
Source: iScience. 2021 Oct 5;24(11):103235. doi: 10.1016/j.isci.2021.103235 (PMC8551077; doi:10.1016/j.isci.2021.103235)
Supplement: Document S1. Figures S1–S7 and Tables S1–S4 [file mmc1.pdf]

iScience, Volume *24*

Supplemental information

Conditions for maintenance of hepatocyte differentiation and function in 3D cultures

Niklas Handin, Evgeniya Mickols, Magnus Ölander, Jakob Rudfeldt, Kristin Blom, Frida Nyberg, Wojciech Senkowski, Jozef Urdzik, Varun Maturi, Mårten Fryknäs, and Per Artursson

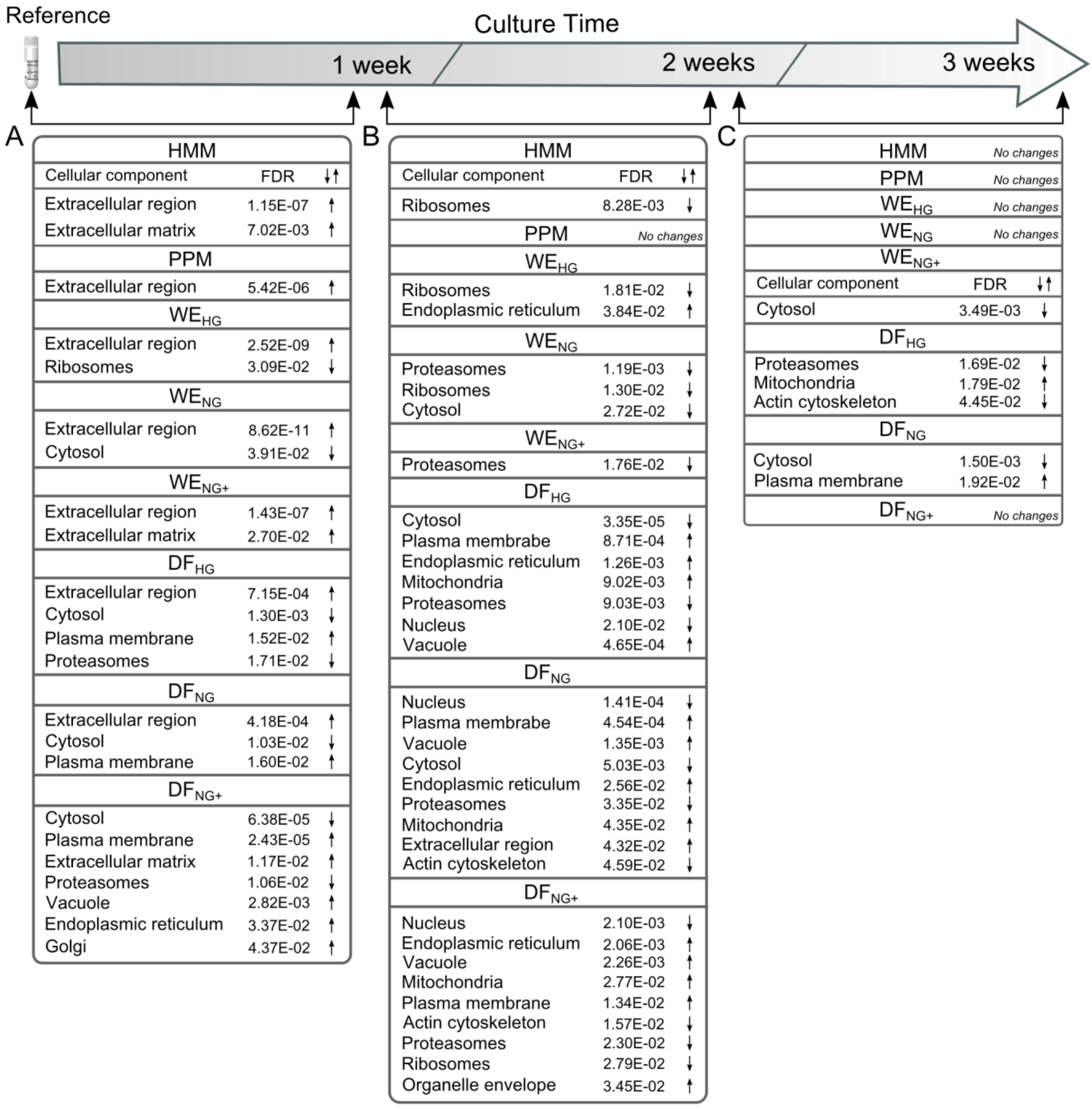

***Fig S1**. **Statistical enrichment analysis of significantly up- and downregulated (indicated by arrows) cellular components between two time points, related to Fig. 4.** Data was collected using quantitative global proteomics on PHH spheroids from four donors. **a**, reference and spheroids cultured for 1 week; **b**, spheroids cultured between 1 and 2 weeks; **c**, spheroids cultured between 2 and 3 weeks. False discovery rate values smaller than 0.05 were considered significant.*

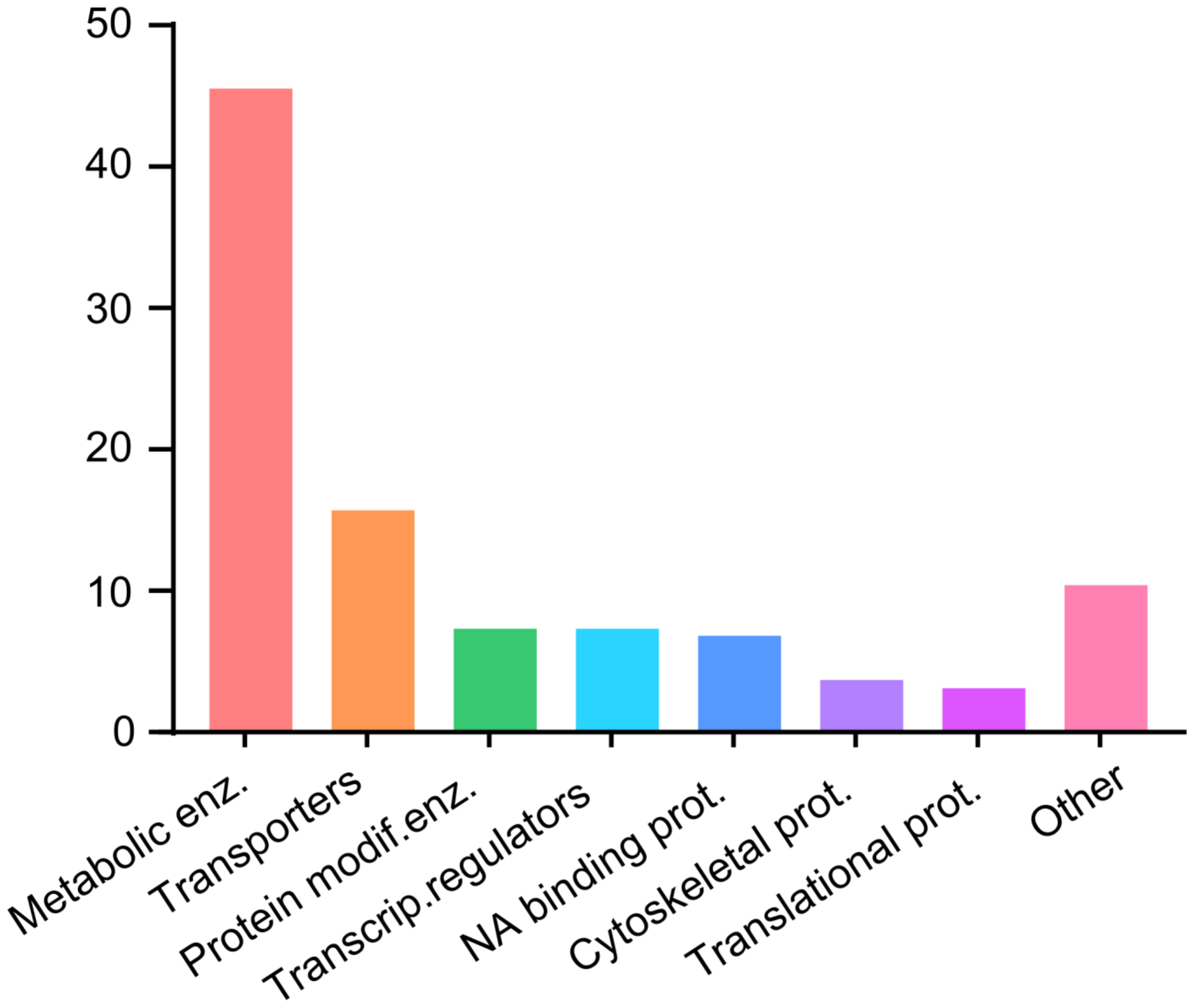

Fig S2. Protein classification of the ADME relevant proteins, related to Fig. 5. *The protein classes of the 315 (227 hits with identified function in Panther) identified ADME-related proteins in the PPH spheroids during three weeks of culture. The y-axis shows the percentage of proteins per class*.

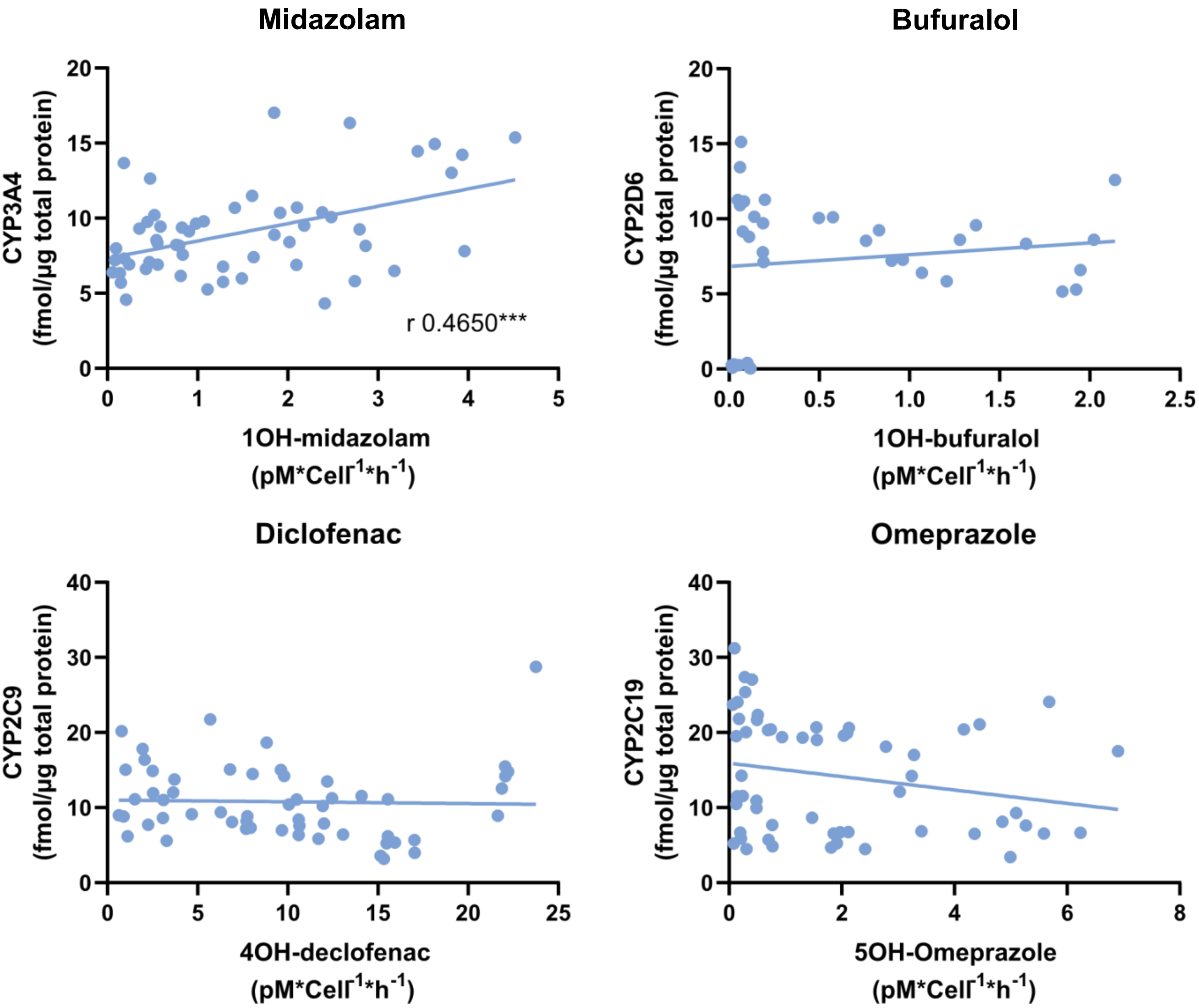

Fig S3. Linear correlation between protein abundance and metabolite formation rate, related to Fig. 6. *The PHH spheroids were incubated with substrates (midazolam, bufuralol, omeprazole and diclofenac). Their primary metabolites (1OH-midazolam, 1OH-bufuralol, 5OH-omeprazole and 5OH-diclofenac) were measured using LC-MS/MS. The PPM medium was excluded in the correlation study as it showed irregular metabolite formation rate. Midazolam showed a significant correlation expressed as Persons correlation coefficient).*

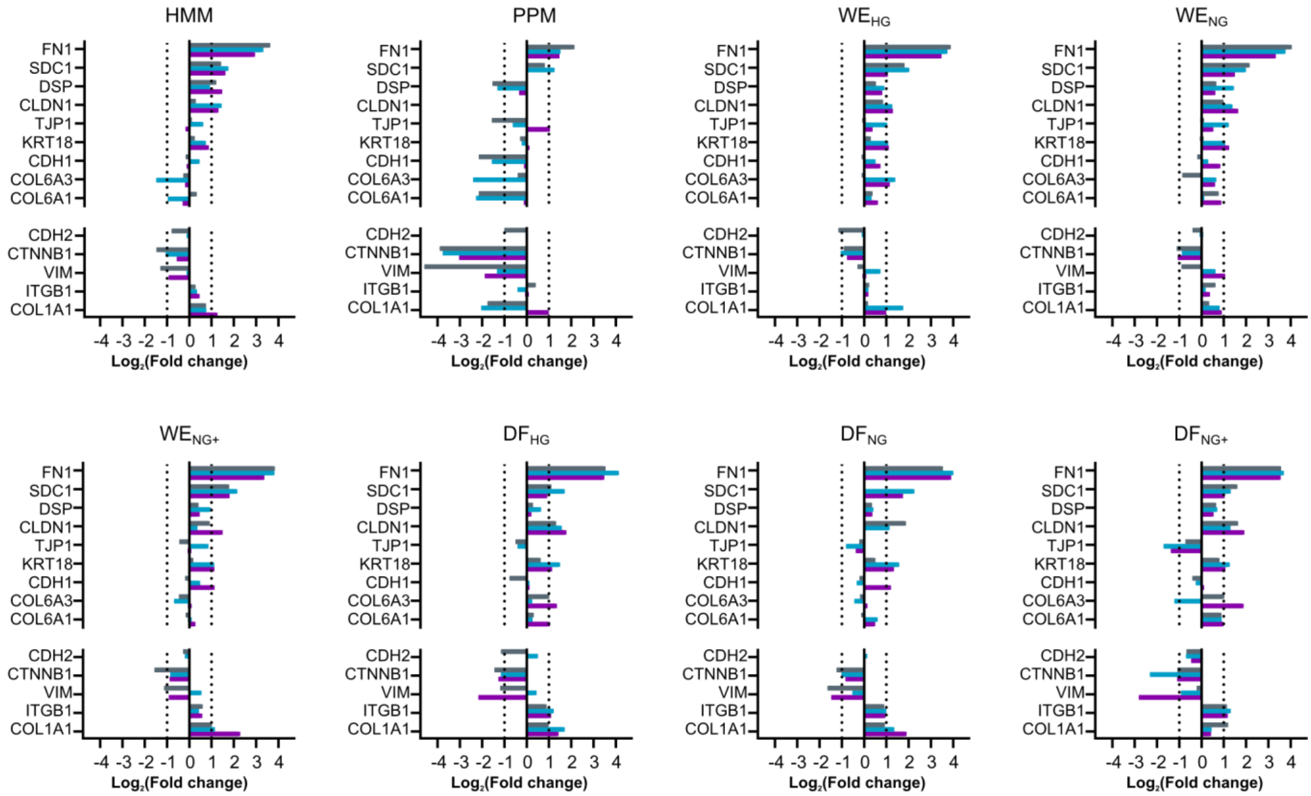

Fig S4 Expression of epithelial and mesenchymal markers in PHH spheroids , related to Fig. 4. *Expression of epithelial (upper) and mesenchymal markers (lower) from PHH spheroids from four donors was compared to the reference (freshly thawed primary human hepatocytes). Measurements were taken at one, two and three weeks of culture. Dotted lines indicate unchanged protein expression (±2-fold over the reference).*

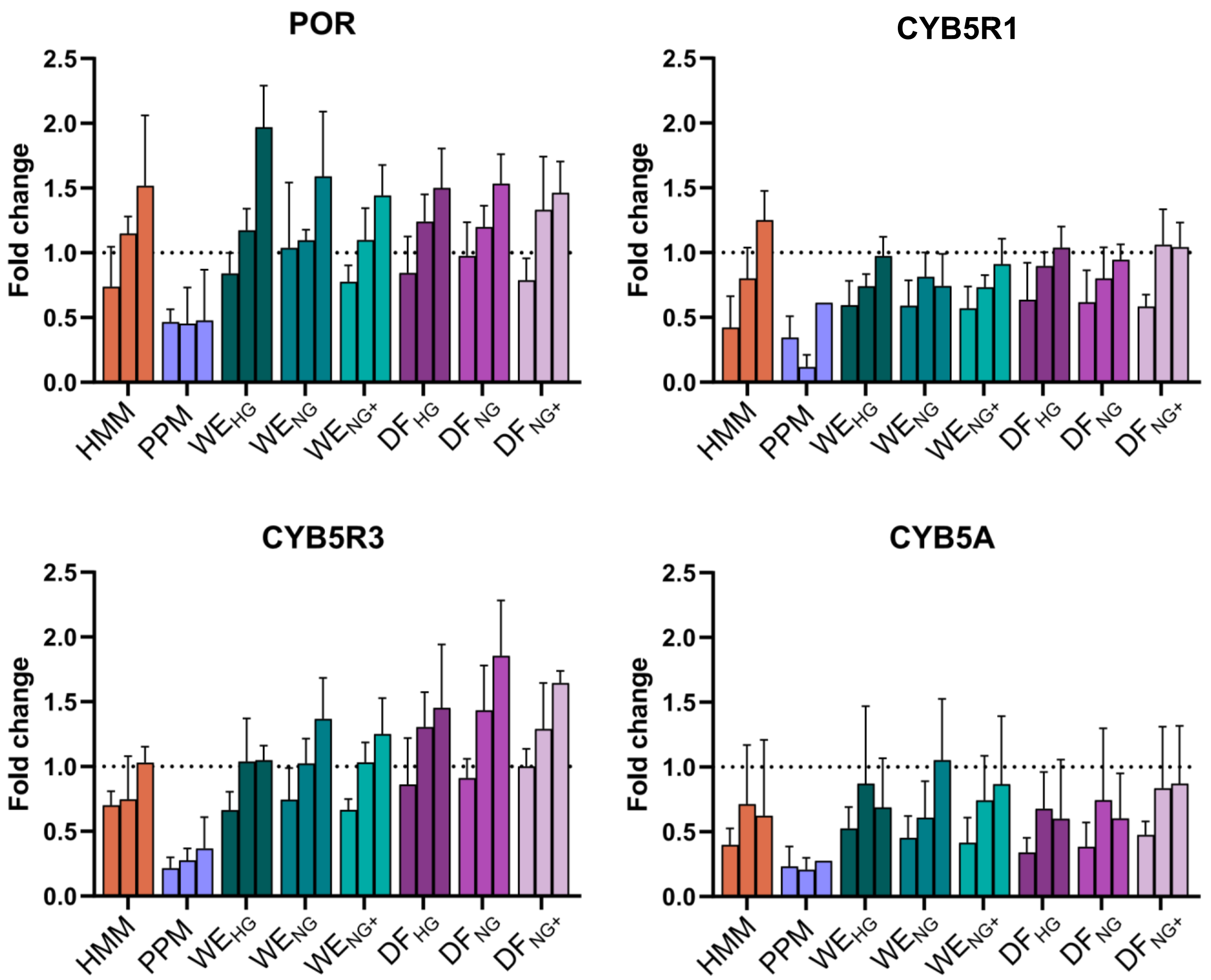

Fig S5. Expression of proteins that acts as electron donors for relevant CYP450 proteins , related to Fig. 6. *Protein expression was measured for PHH spheroids from four donors at one, two and three weeks of culture and compared to freshly thawed PHH for the eight media. Data are represented as average and error bars are showing standard deviation*

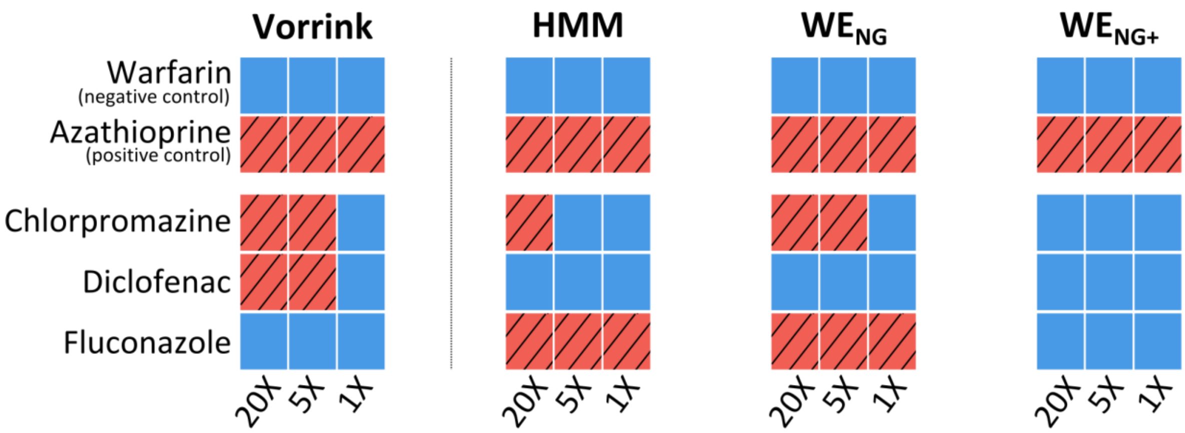

Fig S6. Hepatotoxic evaluation of five drugs in three media, related to Fig. 6. *PHH spheroids from four donors were grown for 7 days in specified media and then incubated with the compounds for 14 days. Compound concentration is 1x, 5x or 20x times their clinical* C_{max}*. Left: results from the literature* (Vorrink et al., 2018)*. Red boxes with stripes indicate that the average hepatocyte viability was decreased to <80% of the respective controls and blue boxes if they did not.*

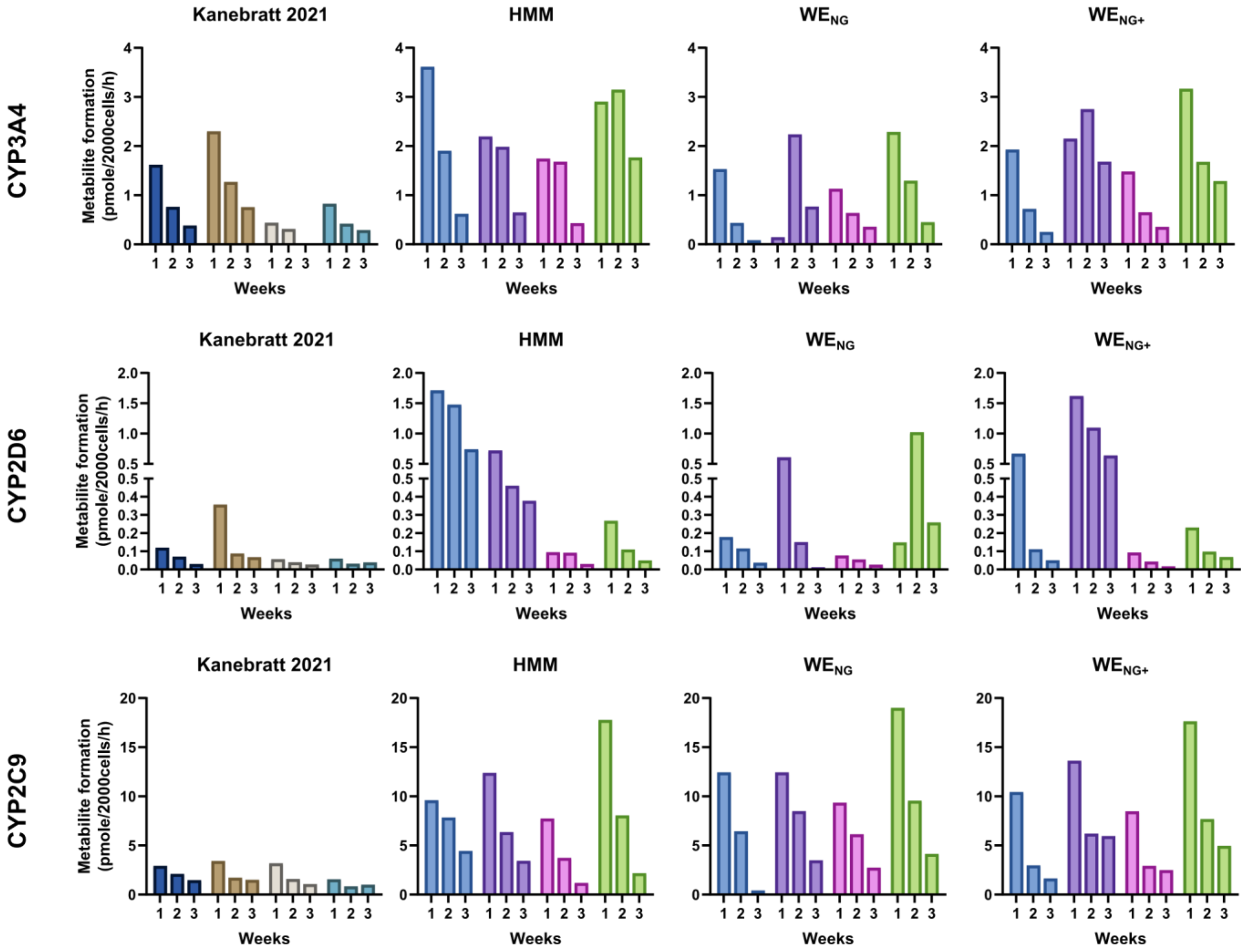

Fig S7. Metabolite formation rate of PHH spheroids from four donors (blue, purple, pink, green) cultured in different media, related to Fig. 6. *PHH spheroids from four donors were cultured for 1, 2 and 3 weeks and subjected to a cocktail of prototypical cytochrome P450 substrates at each time point. Metabolite formation rate was calculated using LC-MS/MS. The left-most graph shows values from the literature* (Kanebratt et al., 2021)*. Note that the units should be pmol/h/spheroid in figure S2A from Kanebratt et al., 2021, as confirmed by the authors.*

Table S1. The transitions, their declustering potential and collision energy, related to Fig. 6.

	Q1	*Q3*	*DP*	*CE*
1OH-bufuralol	278.1	186.1, 159.2	35	26, 36
1OH-midazolam	342.1	203.2, 167.9, 175.9	15	28, 42, 18
5OH-omeprazole	362.1	196.2, 152.5, 121.0	30	38, 44, 44
4OH-declofenac	310.1	265.9, 229.9, 194.0	-15	-17, -13, -27
Warfarin	309.0, 307.0	163.0, 161.0	10, -10	20, -28

Table S2. Cell origins and culture format for albumin secretion comparison , related to Fig. 1.

REFERENCE (DOI)	*CELL ORIGIN*	*CULTURE FORMAT*	*ASSUMPTIONS CALCULATION*
**10.1016/ J.CELL.2018.11.013**	Human liver	Matrigel Organoids	
**10.1002/ LT.22200**	Human liver	PLLA Scaffold spheroids in flow bioreactor	
**10.1002/ HEP.24760**	Human liver	Spheroids in perfusion bioreactor	
**10.1371/ JOURNAL.PONE.0229654**	Human liver	2D plated hepatocytes	675 pg protein/cell and 0.5 mL media in each 24-well plate.
**10.1038/ SREP28178**	Human liver and 3T3-J2 murine embryonic fibroblasts	Micropatterned co-cultures	
**10.1002/ BIT.26341**	Rat hepatocytes	Microfluidic perfusion	
**10.1016/ J.BIOMATERIALS.2015.11.026**	HepaRG	Spheroids	
**JOURNAL? 10.1007/ S00204-014-1215-9**	HepG2 cell	Matrigel Spheroids	50 µL in each well

Table S3. Medium cost at the time of purchase, related to Table. 1.

MEDIUM	*COST (SEK/L)*
**HMM**	8,040
**WILLIAMS E**	868
**PPM**	14,508
**DMEM/F12**	330

Table S4. Donor information, related to Fig. 1-6.

BATCH	*AGE*	*GENDER*	*BMI*	*DIAGNOSIS*
**1**	67	M	28	Colorectal cancer
**2**	77	F	21	Colorectal cancer
**3**	61	F	23	Colorectal cancer
**4**	39	F	20	Breast cancer
